# Factors associated with uptake and acceptability of cervical cancer screening among female sex workers in Northeastern Uganda: A cross-sectional study

**DOI:** 10.1371/journal.pone.0312988

**Published:** 2025-01-24

**Authors:** Ronald Opito, Emmanuel Tiyo Ayikobua, Hellen Akurut, Susan Alwedo, Saadick Mugerwa Ssentongo, Walter Drake Erabu, Lazarus Oucul, Musa Kirya, Lameck Lumu Bukenya, Elly Ekwamu, Abraham Ignatius Oluka, Samuel Kabwigu, Emmanuel Othieno, Amos Deogratius Mwaka

**Affiliations:** 1 Department of Public Health, School of Health Sciences, Soroti University, Soroti, Uganda; 2 Department of Physiology, School of Health Sciences, Soroti University, Soroti, Uganda; 3 Directorate of Program Management and Capacity Development, The AIDS Support Organization, Kampala, Uganda; 4 Department of Health, Kaberamaido District Local Government, Kaberamaido, Uganda; 5 Department of Surgery, School of Health Sciences, Soroti University, Soroti, Uganda; 6 Department of Obstetrics and Gynecology, School of Health Sciences, Soroti University, Soroti, Uganda; 7 Department of Pathology, School of Health Sciences, Soroti University, Soroti, Uganda; 8 Department of Internal Medicine, Faculty of Medicine, Gulu University, Gulu, Uganda; University of Ibadan, NIGERIA

## Abstract

**Background:**

Cervical cancer screening program in Uganda is opportunistic and focuses mainly on women aged 25–49 years. Female sex workers (FSWs) are at increased risk of developing invasive cervical cancer. There is limited data regarding the uptake and acceptability of cervical cancer screening among FSWs in Uganda. This study aimed at identifying factors associated with uptake and acceptability of cervical cancer screening among FSWs in Eastern Uganda.

**Methods:**

This was a cross-sectional study conducted among 423 FSWs aged 18–49 years attending care at six health facilities serving Key Populations (FSWs, Men who have sex with men, transgender people, people who inject drugs and people in prisons) in the Teso sub-region. Data was collected using structured investigator administered questionnaire and analyzed using Stata statistical software version 15.0 (Stata Corp, Texas, USA). The primary outcome was uptake of cervical cancer screening measured as the proportion of female sex workers who have ever been screened for cervical cancer. Chi-square test was used to compare the differences in uptake of cervical cancer screening by HIV status. Modified Poisson regression model with a robust variance estimator was used to determine association between the outcome variables and selected independent variables including demographic characteristics. Prevalence ratios (PR) with accompanying 95% confidence intervals have been reported. Statistical significance was considered at two-sided p-values ≤ 0.05.

**Results:**

The mean age of the participants was 28.1 (±SD = 6.6) years. The self-reported HIV prevalence was 21.5% (n = 91). There were 138 (32.6%) participants who had ever been screened for cervical cancer (uptake), while 397 (93.9%) were willing to be screened (acceptability). There was a significant difference in cervical cancer screening uptake between women living with HIV (WLHIV) and those who were HIV negative, 59.3% vs 26.9% respectively (P < 0.001). The significant factors associated with uptake of cervical cancer screening included living with HIV, adjusted prevalence ratio (aPR) = 1.53 (95%CI: 1.15–2.07), increasing number of biological children, aPR = 1.14 (1.06–1.24) living near a private not for profit (PNFP) facility, aPR = 2.84 (95% CI; 1.68–4.80) and availability of screening services at the nearest health facility, aPR = 1.83 (95% CI, 1.30–2.57). Factors significantly associated with acceptability of cervical cancer screening included being 40 years or older, aPR = 1.22 (95%CI: 1.01–1.47), having a family history of cervical cancer, aPR = 1.05 (1.01–1.10), and living near a PNFP facility, aPR = 1.17 (95% CI, 1.09–1.27) and having ever screened before, aPR = 0.92 (0.86–0.98).

**Conclusion:**

Female sex workers living with HIV are more likely to screen for cervical cancer than the HIV negative clients. Cervical cancer screening uptake is relatively low among the female sex workers. However, majority of the FSWs are willing to be screened for cervical cancer if the services are provided in the nearby healthcare facilities. There is need to make cervical cancer screening services available to all eligible women especially the female sex workers and integrate the services with sexual reproductive health services in general and not just HIV/ART clinics services.

## Introduction

In the low- and middle-income countries (LMICs), cervical cancer is still one of the commonest (second most common) cancers accounting for significant mortality [1]. In Uganda, there were an estimated 6,938 new cervical cancer cases diagnosed, with 4,782 deaths from the disease in 2022, translating into age standardized incidence and mortality of 53.8 and 40.6 per 100,000 women respectively [2].

High risk Human papillomavirus (hrHPV) types16 and 18 are responsible for about 70% of the global new cervical cancer cases [3]. Women living with HIV (WLHIV) are particularly at increased risk of cervical cancer as compared to their HIV negative counterparts [4–6]. Other factors associated with development of cervical cancer and HPV positivity include; early sexual debut [7], history of having a sexually transmitted infection [5,6,8] and having more than 2 lifetime sexual partners [6]. There is a disproportionately high prevalence of HPV and a correspondingly high prevalence of cervical cancer among female sex workers globally [7,8]. Female sex workers are at a high risk of acquiring HPV, HIV and other sexually transmitted infections (STIs) mainly because of having multiple sex partners and having unprotected sex, both of which are facilitators of cervical cancer development [9]. This subpopulation of women therefore requires increased attention to protect them from developing cervical cancer or at least screen them regularly to detect cancer in pre-invasive phase or early stage when treatment can be beneficial.

Cervical cancer can be prevented by using proven cost effective measures including scheduled screening to detect early lesions that can be treated effectively [10]. The global strategy to accelerate the elimination of cervical cancer by 2030 involves the three pillars of achieving: 90% Human papillomavirus (HPV) vaccination coverage of eligible girls, 70% screening coverage with a high-performance test, and 90% of women with a positive screening test or a cervical lesion managed appropriately [3]. The success of screening depends on access and uptake, quality of screening tests, adequacy of follow-up, and prompt diagnosis and treatment of lesions detected [10]. However, coverage of cervical cancer screening services is low in the low income countries (LICs) with an average estimated lifetime screening of 11% compared to 84% in high income countries (HICs) [11]. Community-based studies in rural Uganda showed low screening uptake ranging from 4.8% to 7% [10,12]. Even in circumstances where cervical screening services are available, uptake of screening is low due to several barriers. The barriers to cervical cancer screening include negative individual perceptions and health facility related challenges [10]. Other barriers include not being sure of benefits of screening, fear of screening outcomes [13] and the perceptions that screening is expensive, embarrassing, painful and consumes a lot of time [14].

In Uganda, there are some data regarding uptake and acceptability of cervical screening in the general population. However, there is limited understanding of the factors affecting uptake and acceptability of cervical cancer screening among female sex workers in the country, yet female sex workers are particularly at high risk of developing and progressing to invasive cervical cancer [15]. This subpopulation of women also faces several barriers to cervical cancer screening, due to among others poor physical accessibility because of the highly mobile nature of their trade, they are a hard-to-reach population, and often the opening hours for clinics are not convenient for them [16,17]. This study aimed at determining the level of uptake, acceptability of cervical cancer screening, and associated factors among female sex workers in Northeastern Uganda.

## Materials and methods

### Study design and setting

This was a cross-sectional study conducted in Teso sub-region in Northeastern Uganda. The Teso sub-region comprises 10 districts of Soroti, Amuria, Kapelebyong, Katakwi, Bukedea, Kumi, Ngora, Serere, Kalaki and Kaberamaido and Soroti City, with a total population of 1,819,708 [18]. The HIV prevalence in the region is 4.2% [19]. The total number of health facilities supported by The United States President’s Emergency Plan for AIDS Relief (PEPFAR) through Center for Disease Control and prevention (CDC) to provide high quality HIV and cervical cancer screening services is 106 (1 RRH, 7 Hospitals, 10 HCIV, 62 HCIII, 23 HCII and 3 Special clinics), of which 17 had been designated to provide comprehensive HIV prevention, care and treatment services to key and priority populations (FSWs, MSM, people who inject drugs, people in prisons, uniformed personnel, long distant truck drivers, fisher folks) and had served 2043 female sex workers between October 2021 and March 2022 (https://mets.or.ug/kp-dashboard). These health facilities are categorized and ranked by levels, with Health center III (HCIII) being the lowest supported health facility and regional referral hospital (RRH) being the highest tertiary health facility supported and special clinics being those health facilities specialized in the provision of HIV prevention, care, and treatment services. The estimated population of female sex workers in the region is 5,784 [20]. The central point of the region is Soroti district which is located 326 km Northeast of Uganda’s capital Kampala. Our study purposively selected six (6) high volume health facilities which served at least 100 female sex workers in six months. These included Katakwi hospital, Toroma HCIV, Kumi HCIV, Eastern division HCIII, Kichinjajji HCIII and TASO Soroti Centre of Excellence, which in total had served 1,633 (80%) female sex workers in the 6 months period (October 2021 to March 2022).

### Study population

The study population included all female sex workers 18–49 years of age receiving HIV prevention, care, and treatment services in the Teso sub-region during the study period. We included all female sex workers, who were willing and able to provide voluntary informed consents in the study. We excluded female sex workers who had not yet attained majority age, women with a history of hysterectomy and those who were very ill at the time of data collection.

### Sample size estimation

The sample size was estimated using the Kish Leslie equation (1965) [21]. To get large sample size that can help evaluate several factors associated with uptake, we used uptake prevalence of 50% since there are very scarce study in this subpopulation. We obtained a sample size of 384 and adjusted for 10% non-response to obtain a sample size of 422 participants. In our final analysis, we included 423 participants distributed in the six facilities proportionate to the number of individuals served per facility as follows: Katakwi Hospital (40), Toroma HCIV (27), Kumi HCIV (200), Eastern Division HCIV (30), Kichinjajji HCIII (93) and TASO Soroti Centre of Excellence (33).

### Participants sampling and sampling technique

We used consecutive sampling to recruit the female sex workers coming for HIV prevention and treatment services and willing to participate in the study. We consented and recruited all those who accepted to participate in the study. Recruitment and data collection were conducted during March 2023. Prospective participants were recruited until each site sample size was reached.

### Study variables

The primary outcome variable was uptake of cervical cancer screening among female sex workers. Cervical cancer screening uptake was measured as self-reported ever undertaking of a cervical cancer screening test. Respondents were specifically asked “Have you ever been tested or examined for cervical cancer or precancer?” (No/Yes). The secondary outcome variable was acceptability of cervical cancer screening where participants were asked whether they were willing to accept cervical cancer screening if provided with the opportunity (No/Yes). The independent variables included socio-demographic information including age, religion, occupations, place of residence, ethnicity, family history of cervical cancer, number of sexual partners, marital status, and parity. We also included other health characteristics based on findings from previous studies. These factors included self-reported use of family planning, HIV status, distance to the nearest health facility categorized as less than 5 km and five or more km [22], history of STI diagnosis and treatment in the last one year, and recent visit to health facility.

### Data collection and management

**Fig 1:**
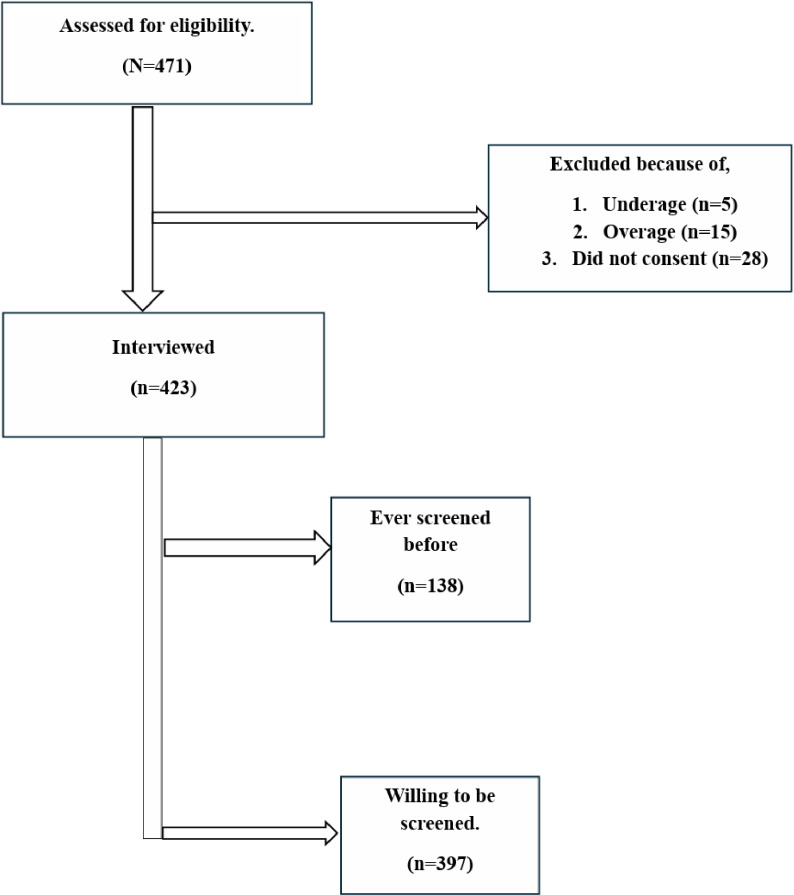
Participant Recruitment flow diagram.

Data was collected using investigator administered questionnaire installed into android phones loaded with the online Kobo toolbox software (https://www.kobotoolbox.org/). We trained ten (10) research assistants (5 female and 5 male) who were medical/nursing students at Soroti University to recruit and interview participants. The research assistants (RA) were trained on the study objectives, matters related to the female sex work, and confidentiality required during research, consenting process, and the interview technique using the kobo toolbox for online data collection. They were also trained on aspects of cervical cancer including the burden, risk factors and symptoms, and the different approaches to prevention, screening and early detection approaches, and management. The RAs approached the clients when they had come to receive clinical care including HIV prevention education, care for sexually transmitted infections (STI), and treatment for other diseases. The RAs explained the purpose and process of the study to the prospective participants. Prospective participants were informed that participation in the study would not in any way influence the services they receive at the facility and that they were free to withdraw from the study at any point they feel like withdrawing. They were allowed to ask questions regarding the study. After their concerns were addressed, the study participants were offered and completed the informed consent forms before interviews. We administered the questionnaire to all eligible study participants that provided informed consent. Fig 1 below shows the recruitment flow chart.

### Data analysis

Data from the Kobo toolbox software was downloaded onto an excel sheet at the end of each day of data collection. RO and AIO reviewed the data for completeness after download and before storage. Incomplete records were completed with help of the RAs and or by calling the participants. Those that could not be completed reliably were removed from the dataset. Duplicated participant unique ID were corrected to ensure each unique ID is assigned to only one participant. RO and AIO cleaned the data, assessed for missing values, and inconsistent entries. The cleaned data was then exported to STATA version 15.0 statistical software (Stata Corp, Texas, USA) for further management including analysis. RO and AIO conducted data analysis. At univariate analysis, the socio-demographics including age, marital status, and duration of sex work was summarized and presented as frequencies, median with interquartile ranges, and mean with standard deviations. Both the primary outcome, uptake of cervical cancer screening, and the secondary outcome, acceptability of cervical cancer screening were presented as proportions. We conducted bivariate analysis between the pre-selected independent predictor variables mentioned above and the two outcome variables. We then used Modified Poisson regression model with a robust variance estimator to determine factors associated with uptake and acceptability of cervical cancer screening among FSWs. Modified Poisson was chosen over logistic regression because the outcome is common (based on similar studies), and we wanted to estimate prevalence ratios instead of odds ratios. Before conducting the multivariate Poisson regression, the pre-selected variables were included in a correlation model and checked for multicollinearity [23]. Where variables showed multicollinearity (r ≥ 0.4), only one was included in the final model. The measures of effect sizes reported are prevalence ratios with their corresponding 95% confidence intervals. Variables with two-sided p-value < 0.05 were considered statistically significant.

### Ethical approval and consent to participate

Ethical clearance for this study was sought from the TASO Research and Ethics Committee (REC), approval number, TASO-2022-151. Administrative clearances were sought from the office of the district health officer (DHO) of the respective districts and permission to conduct the study sought from the in-charges of the facilities where the study was conducted. The individual participant provided written informed consent after the research assistants had explained to them the aims of the study, the benefits and risks associated with the study. Participants’ information was anonymized before entry into the database for analysis. This was to ensure confidentiality and privacy. We reported the results as disaggregated data without revealing individual information.

## Results

### Socio-demographic characteristics

The mean age of the 423 participants was 28.2 (SD = 6.6) years; the majority were young, aged 20 –29 years (54.1%, n = 229), never married (62.9%, n = 266), and with primary level education (44.2%, n = 187). Most participants (70.9%, n = 300) had been in sex work for less than five years, and were current users of hormonal contraceptives (82.5%, n = 349). Majority had no history of previous sexually transmitted infections (61.8%, n = 257). The self-reported HIV prevalence in this study was 21.5% (n = 91). Nearly half of the HIV negative participants (42.6%, n = 130) were on pre-exposure prophylaxis. Most participants (94.6%, n = 400) lived within less than five kilometers to the nearest health facility (Table 1).

**Table 1:** Socio-demographic and health related characteristics of study participants N = 423.

Factors		
**Continuous variables**	**Mean (SD)**	**Median/IQR**
Current age (Years)	28.2 (6.6)	
No of biological children		2 (1–3)
**Categorical Variables**	Frequency (n)	**Proportions (%)**
**Age Category**		
< 20 Years	30	9.1
20–29 Years	229	54.1
30–39 Years	142	33.6
40+ Years	22	5.2
**Marital status**		
Never married (single)	266	62.9
Married	18	4.3
Widow	18	4.3
Divorced/separated	121	28.6
**Duration of sex work**		
< 5 years	300	70.9
≥ 5 years	123	29.1
**Education level**		
No formal education	40	9.5
Primary level	187	44.2
Secondary Level	165	39.0
Tertiary level	31	7.3
**Formal employment**		
Unemployed	193	45.6
Employed	230	54.4
**Cervical cancer screening services is available in the nearest health facility to my residence**		
Yes	289	68.3
No/Not sure	134	31.7
**Distance to nearest health facility (kilo meters)**		
< 5 km	400	94.6
≥ 5 km	23	5.4
**Self-reported HIV status**		
Negative	305	72.1
Positive	91	21.5
Unknown	27	6.4
**Current PrEP User for HIV negative (N = 305)**		
No	175	57.4
Yes	130	42.6
**Using hormonal family planning during study time**		
No	74	17.5
Yes	349	82.5
**Ever been diagnosed with STI in recent one year**		
No	257	61.8
Yes	166	38.2
**Age at first sex**		
< 15 Years	105	24.8
≥ 15 years	318	75.2
**Family history of cervical cancer**		
No	339	80.1
Yes	84	19.9

STI = sexually transmitted infections; PrEP = Pre-exposure prophylaxis.

### Socio-demographic and health systems factors associated with cervical cancer screening uptake

Cervical cancer screening uptake was 32.6% (95%CI; 28.3–37.3), (n = 138). There was a significant difference in cervical cancer screening uptake between women living with HIV and those who were HIV negative, 59.3% vs 26.9% (P < 0.001) respectively. At multivariate level, self-reported cervical cancer screening was independently associated with being HIV positive, adjusted prevalence ratio, aPR = 1.53 (95% CI; 1.15–2.07). Cervical cancer screening uptake increased with increasing number of biological children, aPR = 1.14 (95%CI, 1.06–1.24). Participants who lived near private not for profit (PNFP) health facilities were thrice as likely to have undergone cervical cancer screening than those who lived nearest a public (government owned) facility, aPR = 2.84 (95%CI; 1.68–4.80). Regarding level of health facility, participants living near to a health center IV were less likely to have undergone cervical cancer screening than those living near to a health center III, aPR = 0.63 (95% CI; 0.46–0.87). Availability of cervical cancer screening services in the nearest health facility was statistically significantly associated with uptake of cervical cancer, aPR = 1.83 (95% CI; 1.30–2.57) (Table 2).

**Table 2:** Socio-demographic and health systems factors associated with uptake of cervical cancer screening.

Characteristic	Cervical cancer screening status		Prevalence ratio (PR)	
	Ever screened. n (%), 138 (32.6)	Never Screened n (%), 285 (67.4)	Crude PR (95%CI)	Adjusted PR (95%CI)
**Age (Years)**				
< 20	4 (13.3)	26 (86.7)	1	1
20–29	58 (25.3)	171 (74.7)	1.90 (0.74–4.86)	1.17 (0.47–2.91)
30–39	65 (45.8)	77 (54.2)	**3.43 (1.35–8.71)**	1.36 (0.53–3.54)
≥ 40	11 (50.0)	11 (50.0)	**3.75 (1.37–10.24)**	1.35 (0.49–3.72)
**Marital Status**				
Never married	72 (27.1)	194 (72.9)	1	1
Married	5 (27.9)	13 (72.2)	1.02 (0.47–2.22)	0.79 (0.36–1.72)
Widowed	12 (66.7)	6 (33.3)	**2.46 (1.68–3.61)**	1.03 (0.65–1.63)
Divorced**/**Separated	49 (40.5)	72 (59.5)	**1.50 (1.12–2.01)**	0.96 (0.71–1.29)
**No. of Biological children**				
(per one child)			**1.19 (1.12–1.27)**	**1.14 (1.06–1.24)**
**Educational attainment**				
No formal education	19 (47.5)	21 (52.5)	1	1
Primary	63 (33.7)	124 (66.3)	0.71 (0.48–1.04)	1.01 (0.66–1.53)
Secondary	38 (23.0)	127 (77.0)	**0.48 (0.32–0.74)**	0.82 (0.50–1.33)
Tertiary	18 (58.1)	13 (41.9)	1.22 (0.79–1.90)	1.62 (0.97–2.73)
**Formal employment Status**				
Unemployed	69 (35.7)	124 (64.3)	1	1
Employed	69 (30.0)	161 (70.0)	0.84 (0.64–1.10)	1.17 (0.87–1.57)
**HIV status**				
Negative	82 (26.9)	223 (73.1)	1	1
Positive	54 (59.3)	37 (40.7)	**2.21 (1.71–2.84)**	**1.53 (1.15–2.07)**
**Ever been diagnosed with STI**				
No	84 (32.7)	173 (67.3)	1	1
Yes	54 (32.5)	112 (67.5)	1.00 (0.75–1.32)	1.12 (0.85–1.87)
**There is a family history of cervical cancer**				
No	109 (32.1)	230 (67.9)	1	1
Yes	29 (34.5)	55 (65.5)	1.07 (0.77–1.50)	1.17 (0.86–1.58)
**Distance to nearest facility (Km)**				
< 5	131 (32.8)	269 (67.2)	1	1
≥ 5	7 (30.4)	16 (69.6)	0.93 (0.49–1.75)	1.15 (0.60–2.21)
**Ownership of nearest HF**				
Government	118 (29.7)	279 (70.3)	1	1
Private/PNFP	20 (76.9)	6 (23.1)	**2.59 (2.00–3.35)**	**2.84 (1.68–4.80)**
**Level of Nearest Health facility**				
HCIII	45 (37.8)	75 (62.2)	1	1
HCIV	58 (25.7)	168 (74.3)	**0.68 (0.02–0.49)**	**0.63 (0.46–0.87)**
District Hospital	23 (41.8)	32 (58.2)	1.11 (0.75–1.63)	0.68 (0.41–1.13)
RRH	12 (52.2)	11 (47.8)	1.38 (0.88–2.17)	0.96 (0.57–1.61)
**Cervical cancer screening services available in the nearest facility**				
No/not sure	25 (18.7)	109 (81.3)	1	1
Yes	113 (39.1)	176 (60.1)	**2.10 (1.43 – 3.07)**	**1.83 (1.30–2.57)**

**Bold** = Significant with P < 0.05; All factors were adjusted for each other.

### Acceptability of cervical cancer screening

Most participants, 397 (93.9%; 95%CI, 91.1–95.8) would accept cervical cancer screening if offered. On adjusting the sociodemographic and health systems variables for each other, participants who were 40 years or older, those with a family history of cervical cancer and those residing near a private/PNFP health facilities were more likely to accept cervical cancer screening, with adjusted PRs of 1.22 (95% CI, 1.01–1.47), 1.05 (95% CI; 1.01–1.10) and 1.17 (95%CI; 1.09–1.27) respectively, while participants who had ever screened before were less likely to accept cervical cancer screening, aPR = 0.92 (95%CI; 0.86–0.98) (Table 3).

**Table 3:** Sociodemographic and health systems factors associated with acceptability of cervical cancer screening.

Characteristic	Acceptability cervical cancer screening		Prevalence Ratio (PR)	
	Yes *(n = 397)* N (%)	No *(n = 26)* N (%)	Crude PR (95%CI)	Adjusted PR (95%CI)
**Age Category/years**				
< 20	25 (83.3)	5 (16.7)	1	1
20–29	218 (95.2)	11 (4.8)	1.14 (0.97–1.34)	1.16 (0.99–1.37)
30–39	133 (93.7)	9 (6.3)	1.12 (0.95–1.33)	1.18 (0.99–1.39)
40+	21 (95.5)	1 (4.5)	1.15 (0.95–1.38)	**1.22 (1.01–1.47)**
M**arital status**				
Never married	249 (93.6)	17 (6.4)	1	1
Married	17 (94.4)	1 (5.6)	1.01 (0.90–1.13)	1.00 (0.89–1.11)
Widowed	17 (94.4)	1 (5.6)	1.01 (0.90–1.13)	1.05 (0.92–1.20)
Divorced/Separated	114 (94.2)	7 (5.8)	1.01 (0.95–1.06)	1.00 (0.95–1.05)
**No of Biological Children**				
(Per one child)			1.00 (0.98–1.01)	0.99 (0.97–1.01)
**Education level**				
No formal education	37 (92.5)	3 (7.5)	1	1
Primary	173 (92.5)	14 (7.5)	1.00 (0.91–1.10)	1.00 (0.91–1.11)
Secondary	159 (96.4)	6 (3.6)	1.04 (0.95–1.14)	1.03 (0.93–1.14)
Tertiary	28 (90.3)	3 (9.7)	0.98 (0.84–1.13)	0.98 (0.84–1.15)
**Formal employment**				
Unemployed	176 (91.2)	17 (8.8)	1	1
Employed	221 (96.1)	9 (3.9)	**1.05 (1.00–1.11)**	1.05 (0.99–1.10)
**HIV status**				
Negative	289 (94.8)	16 (5.2)	1	1
Positive	82 (90.1)	9 (9.9)	0.95 (0.88–1.02)	0.96 (0.88–1.03)
**Family history of cervical cancer**				
No	315 (92.9)	24 (7.1)	1	1
Yes	82 (97.6)	2 (2.4)	**1.05 (1.00–1.10)**	**1.05 (1.01–1.10)**
**Cervical cancer screening offered at nearest facility**				
No/Not Sure	128 (95.5)	6 (4.5)	1	1
Yes	269 (93.1)	20 (6.9)	0.97 (0.93–1.02)	1.02 (0.97–1.07)
**Ever Diagnosed with STI**				
No	238 (91.9)	21 (8.1)	1	1
Yes	159 (96.9)	5 (3.9)	**1.06 (1.01–1.10)**	1.04 (0.99–1.09)
**Ownership of nearest Health Facility**				
Government	371 (93.5)	26 (6.5)	1	1
Private/PNFP	26 (100.0)	0 (0.0)	**1.07 (1.04–1.10)**	**1.17 (1.09–1.27)**
**Ever Screened for Cervical cancer**				
No	274 (96.1)	11 (3.9)	1	1
Yes	123 (89.1)	15 (10.9)	**0.93 (0.87–0.99)**	**0.92 (0.86–0.98)**

## Discussion

In this study, most participants were young and attained primary (44%) and secondary (39%) levels of formal education. Most of the participants had never been married or ever got married but divorced/separated. The self-reported prevalence of cervical cancer screening (uptake) was relatively high though still lower than the WHO recommended prevalence of 70% [3]. The acceptability of cervical cancer screening (intention-to-use cervical screening services when availed) was high among the participants. Factors that were statistically significantly associated with uptake of cervical cancer screening included living with HIV, increasing number of biological children, living near to a private not for profit (PNFP) health facility and availability of cervical cancer screening services in the nearest health facility. The factors statistically significantly associated with acceptability of cervical cancer screening were being 40 years or older, having a family history of cervical cancer and residing near a private/PNFP health facilities while participants who had ever screened before were less likely to accept cervical cancer screening.

There were prevalences of risk factors for development of cervical cancer in this study population. For example, the prevalence of self-reported HIV positivity was higher than the regional and national prevalence of 4.2% and 5.8% respectively [19]. The high prevalence of HIV infection among the sex workers in this study is not an isolated finding. HIV prevalence among female sex workers in Uganda is generally higher (33%) than HIV prevalence in the general population [24]. HIV infection is a risk factor for the development and progression of cervical cancer [4–6]. Similarly, the self-reported history of treatment for sexually transmitted infections (STIs) was high among this study participants. And there is evidence that sexually transmitted infections (STIs) including Chlamydia, Genital warts, Syphilis and Gonorrhea are associated with the development of cervical cancer [5,6,8]. In this study, a substantial proportion of participants (25%) reported having started penetrative vaginal sexual intercourse at age < 15 years, while 82.5% of the participants were using hormonal contraceptives. Early sexual debut is a known risk factor for invasive cervical cancer. At such early age, the ability of the immature cervical cells to resist dysplastic changes is still low, and this increases the risks of such women to developing cervical cancer in the future [7,25]. Long term use of hormonal contraceptives especially estrogen containing contraceptives has been found to substantially increase the risk of developing cervical cancer [26]. The female sex workers in this study region, and perhaps other regions of Uganda are exposed to risk factors that may lead to the development of invasive cervical cancer. They therefore need to be encouraged to adopt healthier lifestyles including increased use of condoms, pre-exposure prophylaxis to avoid contracting HIV, regular STI and cervical cancer screening and treatment.

Cervical cancer screening uptake by the female sex workers in this region is generally higher compared to findings from other studies conducted in the general communities in Uganda; prevalence of cervical screening uptake range was 4.8% in eastern region [10], 7.0% in western region [12], and 17.1% to 20.0% in central region [27–29]. Generally, cervical cancer screening uptake is lower in the general community in most countries in sub–Saharan Africa. For example, low cervical cancer screening uptake of 12.9% (95% CI: 10.20, 15.54) was reported in a review of 29 studies on cervical cancer screening uptake in sub–Saharan Africa [30]. Cervical cancer screening uptake is higher among WLHIV attending specialized HIV care compared to screening uptake among women in the general community. A review of studies among WLHIV attending specialized HIV care in Uganda showed that cervical cancer screening uptake ranged between 30% and 59% [13,14]. These findings are comparable to our findings perhaps because the health facilities where we conducted our study are among the facilities supported by TASO to provide specialized HIV prevention, care and cervical cancer screening in the Teso subregion. The facilities also offer health education to clients on matters such as benefits of cervical screening and use of prophylactic ART by female sex workers. The availability of the services in these facilities explains in part the relatively higher cervical cancer screening uptake in our study. Cervical cancer screening uptake prevalence in our study is however lower than uptake in similar populations of female sex workers elsewhere. In Kenya, a study among 418 female sex workers showed that uptake of cervical cancer screening was 72.8% [31]. Similarly, the prevalence of cervical cancer screening uptake was higher among female sex workers (38.6%) in Vancouver, Canada [32]. However, the screening uptake in our study was higher than in a study among 230 sex workers in Ethiopia where the cervical cancer screening uptake prevalence was 13.3% [33].

Female sex workers are a high-risk population that needs deliberate interventions to reduce the incidence and mortality from cervical cancer. These interventions include awareness campaigns to promote behavioral changes such as stopping smoking and adopting cervical cancer screening to reduce their risk of developing the cancer as well as interventions to promote early detection of invasive cervical cancer. Increasing cervical cancer awareness and knowledge about cervical cancer risk factors and symptoms increases screening uptake by about five times [30]. In addition, interventions to increase availability and physical access to health facilities that offer cervical screening services are critical in increasing uptake of cervical screening. In this study, participants nearer to the private not for profit (PNFPs) health facilities that provided cervical cancer screening services were more likely to have been screened. In other studies in Uganda and Jordan, the use of private health care services was a significant positive predictor of cervical cancer screening [34,35]. A scoping review of 19 studies conducted in Africa to determine interventions to increase cervical cancer screening uptake showed that most women were willing to screen before and after the interventions [36]. Lack of cervical screening services is potentially a barrier to cervical cancer screening uptake in sub–Saharan Africa.

There was high acceptability (94%) of cervical cancer screening among this study population. Our finding of high cervical cancer screening acceptability is similar to earlier findings from Mbarara regional referral hospital (65%) in western Uganda [37] and Luwero HCIV (91%) in central Ugandan [38]. However, cervical cancer screening acceptability has varied significantly across regions; in west Africa, studies in Nigeria showed 48% acceptance [39] while in Ghana, acceptance was 82% [40]. The high prevalence of cervical screening acceptability among female sex workers is reassuring. It is therefore appropriate that the Ministry of health of Uganda leverages on the women’s willingness to screen and scale up cervical cancer screening especially in the high-risk population of female sex workers to promote early detection and prompt adequate treatment to minimize morbidity and mortality from cervical cancer.

### Limitations of the study

Our study had some limitations. The study adopted a quantitative survey design that could not lend itself to exploring the nuances regarding the reasons and motivations for the observed cervical screening uptake and acceptability among female sex workers. Such reasons that are necessary to inform design of targeted health interventions to promote cervical cancer screening among female sex workers could best be obtained from exploratory in-depth interviews and or focus group qualitative studies. Second, we based our ascertainment of outcome measures of uptake and acceptability as well as HIV infection status on participants reports which is susceptible to social desirability bias. Third, we estimated sample size using the Kis-Leslie formula for prevalence and did not consider the effect of clustering, which could have potentially affected the power of our study. Introducing a design effect of 1.5 or 2 in calculating sample size could have expanded the sample size and provided us a higher statistical power. We considered the prevalence of 50% to give us the maximum possible sample size for the study and improve on its power. Fourth, our findings ought to be interpreted with cautions since we used consecutive sampling approach to recruit clients into the study; this sampling approach potentially introduces selection and temporal biases. The participants that attended care during the period of the study recruitment can be significantly different in important ways from those that attend in other periods. These biases potentially reduce on representativeness and generalizability of the study findings. However, we are not aware of any unique background interventions or influences that could have differently and systematically driven the sex workers who attended care during the study period. These six health facilities are of varying levels of service provision (lower lever health facilities-HCIIIs, HCIVs, a specialized clinic and a higher-level facility, District hospital). This therefore provided a mix of participants and potentially countered the effects of selection and temporal bias which could have resulted from consecutive sampling.

## Conclusion

Female sex workers are aware of the benefits of screening for cervical cancer and are willing to be screened. However, availability of cervical cancer screening services is limited. The Uganda Ministry of Health may leverage on the women’s willingness to take up cervical cancer screening to scale up the service to promote identification of precancerous lesion in the high-risk population of sex workers, as well as enable early detection of invasive cervical cancer. These will contribute to prevention of invasive cervical cancer and allow for prompt effective treatment of early-stage invasive cervical cancer when treatments can still be effective. Integration of cervical cancer screening and treatment of precancerous lesions with the already established HIV care services is a feasible and sustainable approach to scale up cervical cancer screening and treatment for eligible women especially the female sex workers. There is also need for targeted health education to the female sex workers to promote health lifestyles including avoiding use of alcohol and cigarettes and use of pre-exposure prophylaxis in order to reduce their risks to development of invasive cervical cancer and contracting HIV respectively.

## Supporting information

S1 Data Dataset on uptake and acceptability of cervical cancer screening. (CSV)

S2 Questionnaire Questionnaire uptake and acceptability of cervical cancer screening. (PDF)

## Abbreviations:

AIDS: Acquired immunodeficiency syndrome
CaCx: Cervical cancer
CDC: Centre for Disease Control and Prevention
FSWs: Female Sex Workers
HIV: Human Immunodeficiency virus
HPV: Human Papilloma Virus
KP: Key Populations
REC: Research Ethics Committee
TASO: The AIDS Support Organization
STI: Sexually transmitted infection
VIA: Visual Inspection with acetic acid
WLHIV: Women living with HIV
RRH: Regional Referral Hospital
HC: Health Centre
PEPFAR: President’s Emergency Plan for AIDS Relief.
