# Supplementary material for: Factors associated with uptake and acceptability of cervical cancer screening among female sex workers in Northeastern Uganda: A cross-sectional study
Source: PLoS One. 2025 Jan 24;20(1):e0312988. doi: 10.1371/journal.pone.0312988 (PMC11759397; doi:10.1371/journal.pone.0312988)
Supplement: S2 Questionnaire — (PDF) [file pone.0312988.s002.pdf]

**Appendix V: Participant questionnaire-English**

Objective: To identify factors associated with the utilization and acceptability of cervical cancer screening services among female sex workers in Teso sub-region, Eastern Uganda.

Name of the health facility………………

Level of the health facility………………..

Name and Signature of Research Assistant…………….………….. Date…………..

| **Section 1: Socio-demographic and health system characteristics** | | | |
|---|---|---|---|
| **S/N** | **Question** | | **Response** |
| **Date…………………..** | | **Unique ID………………………………** | |
| 1. | Age in completed years | | …………………. |
| 2 | Current Residence | Village/subcounty | ………………… |
| 3 | Religion | | 1. Catholic<br>2. Muslim<br>3. Protestant<br>4. Pentacostal<br>5. Others (specify) …………. |
| 4 | Marital status (**Tick one appropriate option**) | | 1. never married (single)<br>2. married<br>3. widow<br>4. divorced/separated<br>5. Others specify |
| 5 | How many regular sexual partners are you currently having? | | …………….. |
| 6 | How many sexual clients do you serve per day (on average)? | | ……………….. |
| 7 | How long have you been working as a female sex worker? | | …………………… |
| 8 | How many biological children do you have (parity)? | | ……………….. |

| | | |
|---|---|---|
| 9 | Highest level of education/school completed (**Tick one appropriate option**) | 1. No formal education<br>2. Primary level<br>3. Secondary Level<br>4. Tertiary level<br>5. Vocational |
| 10. | What is your current formal employment status? | 1. Unemployed<br>2. Employed. |
| 11 | If employed, which kind of job | 1. Civil servant (teacher, health worker etc)<br>2. Private Sector employee<br>3. Self-employee/business<br>4. Informal sector employee (eg, porter, cleaner, driver etc). |
| 12 | What is your source of hospital income when sick/visiting a hospital? (tick against the correct response) | 1. From my salary/business<br>2. Donation from friends<br>3. Support from children/relatives<br>4. Use free government services<br>5. Others (specify)…… |
| 13. | Distance in KM of the nearest health facility/hospital from participant's home. (to use google maps to estimate) | ……………………………… |
| 14. | Who owns the nearest health facility? | 1. Government<br>2. Private clinic/hospital<br>3. Private not for profit |
| 15. | Level of that nearest health facility to participant's home. | 1. Health Centre II (Level II)<br>2. Health Centre III (Level III)<br>3. Health center IV (Level IV)<br>4. District hospital (Level V)<br>4. Regional Referral (Level VI) |

| | | |
|---|---|---|
| 16 | Does the nearest health facility provide cervical cancer screening services? | 1. No<br>2. Yes |
| **Section 2: General health and lifestyle practices:** | | |
| 17 | Do you currently smoke cigarette? | 1. No<br>2. Yes |
| 18 | Have you ever smoked cigarette? | 1. No<br>2. Yes |
| 19 | If yes to Qns 15 & 16, for how long (years) have you smoked/been smoking cigarette? | ……………… |
| 20 | Do you currently drink alcohol? | 1. No<br>2. Yes |
| 21 | Have you ever drunk alcohol? | 1. No<br>2. Yes |
| 22 | If yes to Qns 19 or 20, for how long (years) have you drunk/been drinking alcohol? | …………………… |
| 23 | Have you ever used any family method (are you currently using any FP)? | 1. No<br>2. Yes |
| 24 | If yes, which one? | 1. COC/Pills<br>2. Implants<br>3. Depo<br>4. Tubal legation<br>5. IUD<br>6. Others (specify)…. |
| 25 | What is your HIV status? | 1. Negative<br>2. Positive<br>3. Unknown |
| 26 | If HIV positive, are you on medication? | 1. No<br>2. Yes |

| | | |
|---|---|---|
| 27 | If HIV negative, are you on Pre-Exposure Prophylaxis (PrEP)? | 1. No<br>2. Yes |
| 28 | Have you ever been diagnosed and treated for sexually transmitted infections? | 1. No<br>2. Yes |
| 29 | If yes, how many times in the last 3 years? | ……………… |
| 30 | When did you have a thorough medical checkup done? | 1. <6months ago<br>2. 6mo -<1 year ago<br>3. 1-<2 years ago<br>4. 2-<5 years ago<br>5. ≥5 years ago |
| 31 | At what age did you have your first sexual intercourse? | ……………… |
| **32.** | **Have you ever been screened for cervical cancer?** | **1. No**<br>**2. Yes** |
| | Jump qns 33-36 if response to Qn32 was 1 (No), and indicate N/A on them. | |
| 33. | If ever screened for cervical cancer, how long ago? | ……………………………<br>N/A |
| 34 | If ever screened, which method was used? | 1. HPV test<br>2. VIA test<br>3. PAP smear<br>4. I don't Know/remember<br>5. N/A |
| 35 | If ever screened, what was the result? | 1. Negative<br>2. Positive<br>3. I don't remember<br>4. N/A |

| | | |
|---|---|---|
| 36 | If you were screened positive, were you treated? | 1. No<br>2. Yes<br>3. I don’t remember |
| 37. | If never screened, why? | 1. Never been told about CC<br>2. Health workers here don’t screen<br>3. Long distance to health center<br>4. No money for screening<br>5. Others (specify)…………… |
| 38. | Is there any of your family member (Mother or aunties) who are having CC or have died of it? | 1. No<br>2. Yes |
| **39** | **If given a screening opportunity, would you accept to be screened for CC? (acceptability)** | **1. No**<br>**2. Yes** |
| 40. | If you are to be screened, which method would you prefer? | 1. VIA<br>2. HPV<br>3. PAP Smear.<br>4. None |
| **Section 3: Post screening actions (For participants who accept to take up HPV screening).** | | |
| 41 | Was patient screened for CC using HPV test? | 1. No<br>2. Yes |
| 42 | If not screened, why? | 1. Patient choice<br>2. In her menses<br>3. Pregnant<br>4. Just delivered <6weeks. |
| 43 | If yes, result of the screening? | 1. Neg<br>2. Pos |

| | | |
|---|---|---|
| 44 | If positive, HPV subtype detected | 1. 16<br>2. 18<br>3. 45<br>4. Others |
| 45 | If positive, did the patient accept to be referred for treatment? | 1. No<br>2. Yes |
| 46 | If Positive, has patient been referred for further assessment and treatment? | 1. No<br>2. Yes |
